# Crystal structure of 2-(5-meth­oxy-1-benzo­furan-3-yl)acetic acid

**DOI:** 10.1107/S2056989015023609

**Published:** 2015-12-16

**Authors:** Ramakrishna Gowda, K. V. Arjuna Gowda, M. Keshava Reddy, Mahantesha Basanagouda

**Affiliations:** a Department of Physics, Govt. College for Women, Kolar 563 101, Karnataka, India; b Department of Physics, Govt. College for Women, Mandya 571 401, Karnataka, India; c Department of Physics, Govt. PU College, Jayanagara, Bangalore 560 011, Karnataka, India; d Department of Chemistry, P.C. Jabin Science College, Hubli 580 031, Karnataka, India

**Keywords:** crystal structure, benzo­furan, hydrogen bonding

## Abstract

The benzo­furan residue in the title compound, C_11_H_10_O_4_, is essentially planar (the r.m.s. deviation for the nine non-H atoms = 0.011 Å). While the meth­oxy group is coplanar with the fused ring system [C—C—O—C torsion angle = 3.1 (3)°], the acetic acid residue occupies a position almost prime [C—C—C—C = 77.0 (2)°]. In the crystal, centrosymmetrically related mol­ecules are linked by O—H⋯O hydrogen bonds to form eight-membered {⋯HOCO}_2_ synthons. The dimeric aggregates assemble into supra­molecular layers in the *ab* plane *via* benzene-C—H⋯O(ring) inter­actions.

## Related literature   

For a related structures and background to benzo­furans and their applications, see: Dawood (2013 ▸); Khanam & Shamsuzzaman (2015 ▸); Radadiya & Shah (2015 ▸); Naik *et al.* (2015 ▸); Nevagi *et al.* (2015 ▸). For the synthesis, see: Basanagouda *et al.* (2015 ▸).
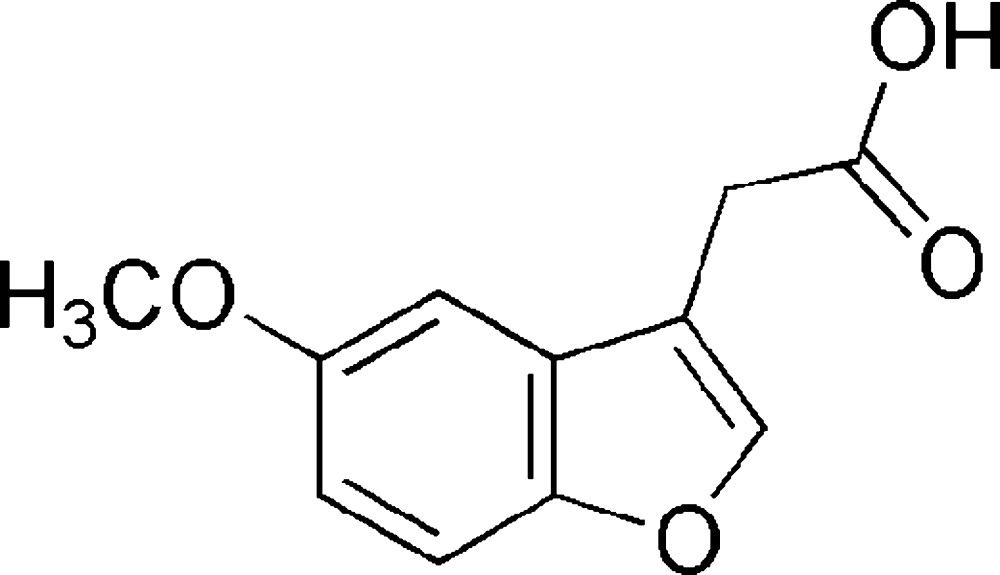


## Experimental   

### Crystal data  

C_11_H_10_O_4_
*M* *_r_* = 206.19
Monoclinic, 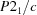

*a* = 5.8096 (3) Å
*b* = 13.2034 (5) Å
*c* = 12.5738 (6) Å
β = 97.641 (3)°
*V* = 955.93 (8) Å^3^
*Z* = 4
Mo *K*α radiation
μ = 0.11 mm^−1^
*T* = 296 K
0.35 × 0.30 × 0.25 mm

### Data collection  

Bruker Kappa APEXII CCD diffractometer
Absorption correction: multi-scan (*SADABS*; Bruker, 2004 ▸) *T*_min_ = 0.961, *T*_max_ = 0.979
12813 measured reflections
2094 independent reflections
1621 reflections with *I* > 2σ(*I*)
*R*_int_ = 0.024

### Refinement  

*R*[*F* ^2^ > 2σ(*F* ^2^)] = 0.040
*wR*(*F* ^2^) = 0.110
*S* = 1.12
2094 reflections
137 parameters
H-atom parameters constrained
Δρ_max_ = 0.22 e Å^−3^
Δρ_min_ = −0.16 e Å^−3^

### 

Data collection: *APEX2* (Bruker, 2004 ▸); cell refinement: *APEX2* and *SAINT* (Bruker, 2004 ▸); data reduction: *SAINT* and *XPREP* (Bruker, 2004 ▸); program(s) used to solve structure: *SIR92* (Altomare *et al.*, 1994 ▸); program(s) used to refine structure: *SHELXL2014* (Sheldrick, 2015 ▸); molecular graphics: *ORTEP-3 for Windows* (Farrugia, 2012 ▸) and *Mercury* (Bruno *et al.*, 2002 ▸); software used to prepare material for publication: *SHELXL2014*.

## Supplementary Material

Crystal structure: contains datablock(s) I, New_Global_Publ_Block. DOI: 10.1107/S2056989015023609/tk5414sup1.cif

Structure factors: contains datablock(s) I. DOI: 10.1107/S2056989015023609/tk5414Isup2.hkl

Supporting information file. DOI: 10.1107/S2056989015023609/tk5414Isup3.cml

**Fig. 1.**
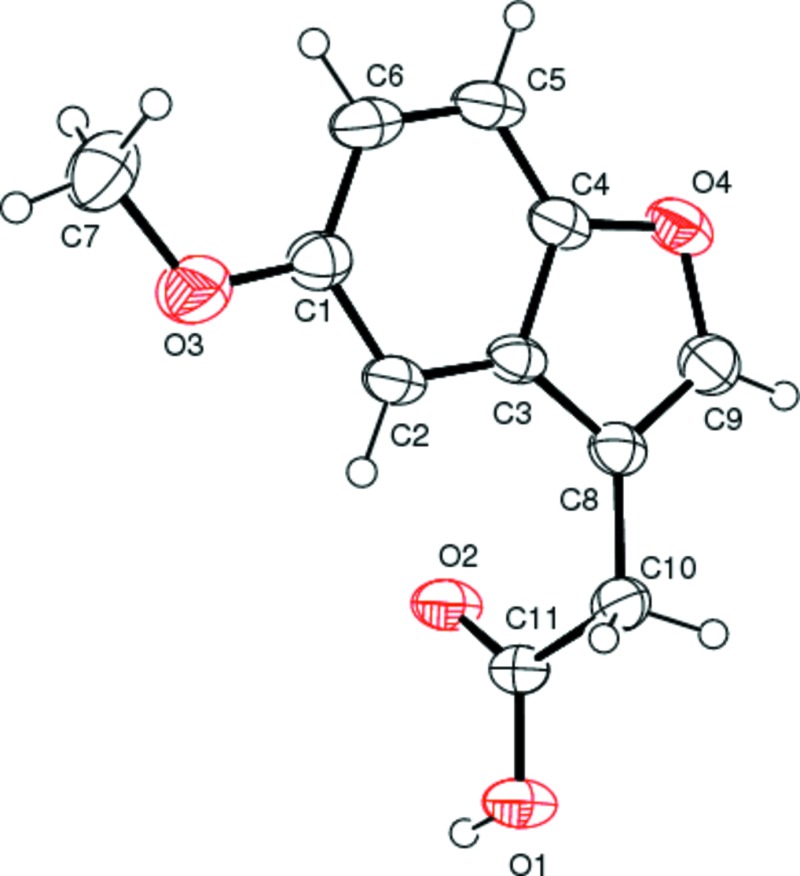
Molecular structure of the title compound showing atom labelling and 40% probability displacement ellipsoids.

. DOI: 10.1107/S2056989015023609/tk5414fig1.tif
Mol­ecular structure of the title compound showing atom labelling and 40% probability displacement ellipsoids.

CCDC reference: 1401314

Additional supporting information:  crystallographic information; 3D view; checkCIF report

## Figures and Tables

**Table 1:** Hydrogen-bond geometry (Å, °)

*D*—H⋯*A*	*D*—H	H⋯*A*	*D*⋯*A*	*D*—H⋯*A*
O1—H1⋯O2^i^	0.82	1.82	2.6357 (17)	174
C2—H2⋯O4^ii^	0.93	2.55	3.4629 (19)	169

Symmetry codes: (i) 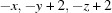; (ii) 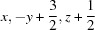.

## supplementary crystallographic information

### S1. Comment

Benzofuran scaffolds have drawn considerable attention due to their physiological and chemotherapeutic properties as well as their widespread occurrence in nature. They display potent biological properties including antihyperglycemic, analgesic, antiparasitic, antimicrobial, antitumor and kinase inhibitor activities (Dawood, 2013; Khanam & Shamsuzzaman, 2015; Radadiya & Shah, 2015; Naik *et al.* 2015; Nevagi *et al.* 2015). In addition, substituted benzofurans find application such as fluorescent sensors, oxidant, antioxidants and brightening agents. The derivatives of 2,3-dihydro-benzofuranyl-3-acetic acid have been reported to be potent, selective and orally bioavailable G protein-coupled receptor 40 (GPR40) and free fatty acid receptor 1 agonists (FFA1) (Basanagouda *et al.*, 2015). A perspective view of the molecule is shown in Fig. 1 and geomtric data for the intermolecular interactions are listed in Table 1.

### S2. Experimental

6-Methoxy-4-bromomethylcoumarin (10 m*M*) was refluxed in 1 *M* NaOH (100 ml) for 2 h (monitored by TLC). The reaction mixture was cooled, neutralized with 1 *M* HCl and the obtained product was filtered off and dried. Colourless blocks were obtained by recrystallization from ethanol and ethyl acetate mixture by slow evaporation.

### S3. Refinement

The carbon-bound H-atoms were placed in calculated positions (C—H = 0.93–0.97 Å) and were included in the refinement in the riding model approximation, with *U*_iso_(H) set to 1.2–1.5*U*_equiv_(C). The oxygen-bound H-atom was also placed in a calculated position (O—H = 0.82 Å) with *U*_iso_(H) set to 1.5*U*_equiv_(O).

### Crystal data

**Table:**

C_11_H_10_O_4_	*D*_x_ = 1.433 Mg m^−^^3^
*M**_r_* = 206.19	Melting point: 413 K
Monoclinic, *P*2_1_/*c*	Mo *K*α radiation, λ = 0.71073 Å
*a* = 5.8096 (3) Å	Cell parameters from 5229 reflections
*b* = 13.2034 (5) Å	θ = 2.2–28.6°
*c* = 12.5738 (6) Å	µ = 0.11 mm^−^^1^
β = 97.641 (3)°	*T* = 296 K
*V* = 955.93 (8) Å^3^	Block, colourless
*Z* = 4	0.35 × 0.30 × 0.25 mm
*F*(000) = 432	

### Data collection

**Table:**

Bruker Kappa APEXII CCD diffractometer	2094 independent reflections
Radiation source: fine-focus sealed tube	1621 reflections with *I* > 2σ(*I*)
Graphite monochromator	*R*_int_ = 0.024
ω and φ scan	θ_max_ = 27.0°, θ_min_ = 2.3°
Absorption correction: multi-scan (*SADABS*; Bruker, 2004)	*h* = −7→7
*T*_min_ = 0.961, *T*_max_ = 0.979	*k* = −16→16
12813 measured reflections	*l* = −16→16

### Refinement

**Table:**

Refinement on *F*^2^	Hydrogen site location: inferred from neighbouring sites
Least-squares matrix: full	H-atom parameters constrained
*R*[*F*^2^ > 2σ(*F*^2^)] = 0.040	*w* = 1/[σ^2^(*F*_o_^2^) + (0.0364*P*)^2^ + 0.4069*P*] where *P* = (*F*_o_^2^ + 2*F*_c_^2^)/3
*wR*(*F*^2^) = 0.110	(Δ/σ)_max_ < 0.001
*S* = 1.12	Δρ_max_ = 0.22 e Å^−^^3^
2094 reflections	Δρ_min_ = −0.16 e Å^−^^3^
137 parameters	Extinction correction: *SHELXL2014* (Sheldrick, 2015), Fc^*^=kFc[1+0.001xFc^2^λ^3^/sin(2θ)]^-1/4^
0 restraints	Extinction coefficient: 0.017 (3)

### Special details

**Table:**

Geometry. All e.s.d.'s (except the e.s.d. in the dihedral angle between two l.s. planes) are estimated using the full covariance matrix. The cell e.s.d.'s are taken into account individually in the estimation of e.s.d.'s in distances, angles and torsion angles; correlations between e.s.d.'s in cell parameters are only used when they are defined by crystal symmetry. An approximate (isotropic) treatment of cell e.s.d.'s is used for estimating e.s.d.'s involving l.s. planes.

### Fractional atomic coordinates and isotropic or equivalent isotropic displacement parameters (Å^2^)

**Table:**

	*x*	*y*	*z*	*U*_iso_*/*U*_eq_	
C1	0.4010 (3)	0.61606 (13)	0.80077 (14)	0.0432 (4)	
C2	0.2178 (3)	0.67578 (12)	0.82316 (13)	0.0389 (4)	
H2	0.1770	0.6787	0.8922	0.047*	
C3	0.0964 (3)	0.73116 (11)	0.74025 (12)	0.0349 (4)	
C4	0.1631 (3)	0.72515 (13)	0.63818 (13)	0.0398 (4)	
C5	0.3460 (3)	0.66724 (14)	0.61521 (14)	0.0477 (5)	
H5	0.3882	0.6653	0.5464	0.057*	
C6	0.4647 (3)	0.61208 (14)	0.69782 (15)	0.0479 (5)	
H6	0.5890	0.5716	0.6848	0.057*	
C7	0.7098 (4)	0.50545 (17)	0.8728 (2)	0.0646 (6)	
H7A	0.7685	0.4733	0.9394	0.097*	
H7B	0.6680	0.4547	0.8190	0.097*	
H7C	0.8272	0.5488	0.8506	0.097*	
C8	−0.0997 (3)	0.79821 (12)	0.73082 (13)	0.0378 (4)	
C9	−0.1369 (3)	0.82596 (14)	0.62760 (14)	0.0476 (4)	
H9	−0.2559	0.8692	0.5995	0.057*	
C10	−0.2391 (3)	0.82719 (13)	0.81742 (14)	0.0421 (4)	
H10A	−0.2708	0.7666	0.8566	0.051*	
H10B	−0.3869	0.8541	0.7845	0.051*	
C11	−0.1270 (3)	0.90353 (12)	0.89552 (13)	0.0367 (4)	
O1	−0.2484 (2)	0.92135 (9)	0.97334 (10)	0.0491 (4)	
H1	−0.1813	0.9636	1.0141	0.074*	
O2	0.0578 (2)	0.94412 (10)	0.88700 (10)	0.0514 (4)	
O3	0.5123 (3)	0.56354 (12)	0.88677 (12)	0.0665 (4)	
O4	0.0193 (2)	0.78381 (10)	0.56745 (9)	0.0503 (4)	

### Atomic displacement parameters (Å^2^)

**Table:**

	*U*^11^	*U*^22^	*U*^33^	*U*^12^	*U*^13^	*U*^23^
C1	0.0431 (9)	0.0419 (9)	0.0459 (10)	−0.0026 (7)	0.0101 (8)	−0.0053 (7)
C2	0.0428 (9)	0.0434 (9)	0.0328 (8)	−0.0051 (7)	0.0133 (7)	−0.0068 (7)
C3	0.0381 (8)	0.0356 (8)	0.0329 (8)	−0.0099 (7)	0.0113 (6)	−0.0089 (6)
C4	0.0456 (9)	0.0428 (9)	0.0325 (8)	−0.0113 (7)	0.0114 (7)	−0.0072 (7)
C5	0.0537 (11)	0.0543 (10)	0.0394 (9)	−0.0101 (9)	0.0218 (8)	−0.0140 (8)
C6	0.0464 (10)	0.0477 (10)	0.0535 (11)	−0.0024 (8)	0.0215 (8)	−0.0139 (8)
C7	0.0515 (12)	0.0578 (12)	0.0832 (15)	0.0092 (10)	0.0038 (11)	−0.0052 (11)
C8	0.0383 (9)	0.0399 (8)	0.0360 (8)	−0.0087 (7)	0.0075 (7)	−0.0069 (7)
C9	0.0477 (10)	0.0514 (10)	0.0439 (10)	−0.0038 (8)	0.0065 (8)	−0.0018 (8)
C10	0.0363 (9)	0.0472 (9)	0.0438 (9)	−0.0026 (7)	0.0093 (7)	−0.0071 (7)
C11	0.0408 (9)	0.0362 (8)	0.0349 (8)	0.0018 (7)	0.0118 (7)	−0.0002 (6)
O1	0.0558 (8)	0.0503 (7)	0.0461 (7)	−0.0115 (6)	0.0248 (6)	−0.0119 (6)
O2	0.0507 (8)	0.0599 (8)	0.0476 (7)	−0.0158 (6)	0.0207 (6)	−0.0172 (6)
O3	0.0661 (9)	0.0760 (10)	0.0590 (9)	0.0257 (8)	0.0144 (7)	0.0081 (7)
O4	0.0604 (8)	0.0598 (8)	0.0322 (6)	−0.0064 (6)	0.0114 (6)	−0.0009 (5)

### Geometric parameters (Å, º)

**Table:**

C1—O3	1.372 (2)	C7—H7A	0.9600
C1—C2	1.383 (2)	C7—H7B	0.9600
C1—C6	1.394 (2)	C7—H7C	0.9600
C2—C3	1.387 (2)	C8—C9	1.338 (2)
C2—H2	0.9300	C8—C10	1.491 (2)
C3—C4	1.391 (2)	C9—O4	1.374 (2)
C3—C8	1.435 (2)	C9—H9	0.9300
C4—C5	1.371 (2)	C10—C11	1.495 (2)
C4—O4	1.375 (2)	C10—H10A	0.9700
C5—C6	1.377 (3)	C10—H10B	0.9700
C5—H5	0.9300	C11—O2	1.217 (2)
C6—H6	0.9300	C11—O1	1.3014 (18)
C7—O3	1.410 (2)	O1—H1	0.8200
			
O3—C1—C2	115.00 (15)	O3—C7—H7C	109.5
O3—C1—C6	123.86 (17)	H7A—C7—H7C	109.5
C2—C1—C6	121.13 (17)	H7B—C7—H7C	109.5
C1—C2—C3	118.41 (15)	C9—C8—C3	105.85 (15)
C1—C2—H2	120.8	C9—C8—C10	127.22 (17)
C3—C2—H2	120.8	C3—C8—C10	126.89 (15)
C2—C3—C4	119.14 (15)	C8—C9—O4	112.94 (17)
C2—C3—C8	134.93 (14)	C8—C9—H9	123.5
C4—C3—C8	105.92 (15)	O4—C9—H9	123.5
C5—C4—O4	126.81 (15)	C8—C10—C11	114.92 (14)
C5—C4—C3	123.03 (17)	C8—C10—H10A	108.5
O4—C4—C3	110.16 (15)	C11—C10—H10A	108.5
C4—C5—C6	117.44 (15)	C8—C10—H10B	108.5
C4—C5—H5	121.3	C11—C10—H10B	108.5
C6—C5—H5	121.3	H10A—C10—H10B	107.5
C5—C6—C1	120.83 (17)	O2—C11—O1	123.98 (15)
C5—C6—H6	119.6	O2—C11—C10	123.47 (14)
C1—C6—H6	119.6	O1—C11—C10	112.55 (14)
O3—C7—H7A	109.5	C11—O1—H1	109.5
O3—C7—H7B	109.5	C1—O3—C7	118.88 (16)
H7A—C7—H7B	109.5	C9—O4—C4	105.13 (13)
			
O3—C1—C2—C3	−179.94 (15)	C4—C3—C8—C9	0.53 (18)
C6—C1—C2—C3	0.7 (3)	C2—C3—C8—C10	−1.0 (3)
C1—C2—C3—C4	−0.3 (2)	C4—C3—C8—C10	178.12 (15)
C1—C2—C3—C8	178.71 (17)	C3—C8—C9—O4	−0.6 (2)
C2—C3—C4—C5	−0.5 (2)	C10—C8—C9—O4	−178.15 (15)
C8—C3—C4—C5	−179.78 (15)	C9—C8—C10—C11	−105.9 (2)
C2—C3—C4—O4	178.93 (14)	C3—C8—C10—C11	77.0 (2)
C8—C3—C4—O4	−0.33 (17)	C8—C10—C11—O2	4.0 (3)
O4—C4—C5—C6	−178.48 (16)	C8—C10—C11—O1	−175.80 (15)
C3—C4—C5—C6	0.9 (3)	C2—C1—O3—C7	−176.25 (17)
C4—C5—C6—C1	−0.4 (3)	C6—C1—O3—C7	3.1 (3)
O3—C1—C6—C5	−179.65 (17)	C8—C9—O4—C4	0.38 (19)
C2—C1—C6—C5	−0.3 (3)	C5—C4—O4—C9	179.42 (17)
C2—C3—C8—C9	−178.54 (18)	C3—C4—O4—C9	−0.01 (18)

### Hydrogen-bond geometry (Å, º)

**Table:**

*D*—H···*A*	*D*—H	H···*A*	*D*···*A*	*D*—H···*A*
O1—H1···O2^i^	0.82	1.82	2.6357 (17)	174
C2—H2···O4^ii^	0.93	2.55	3.4629 (19)	169

Symmetry codes: (i) −*x*, −*y*+2, −*z*+2; (ii) *x*, −*y*+3/2, *z*+1/2.
