# Supplementary figures and images for: Crystal structure of 2-(5-meth­oxy-1-benzo­furan-3-yl)acetic acid
Source: Acta Crystallogr E Crystallogr Commun. 2015 Dec 16;71(Pt 12):o1053–4. doi: 10.1107/S2056989015023609 (PMC4719975; doi:10.1107/S2056989015023609)

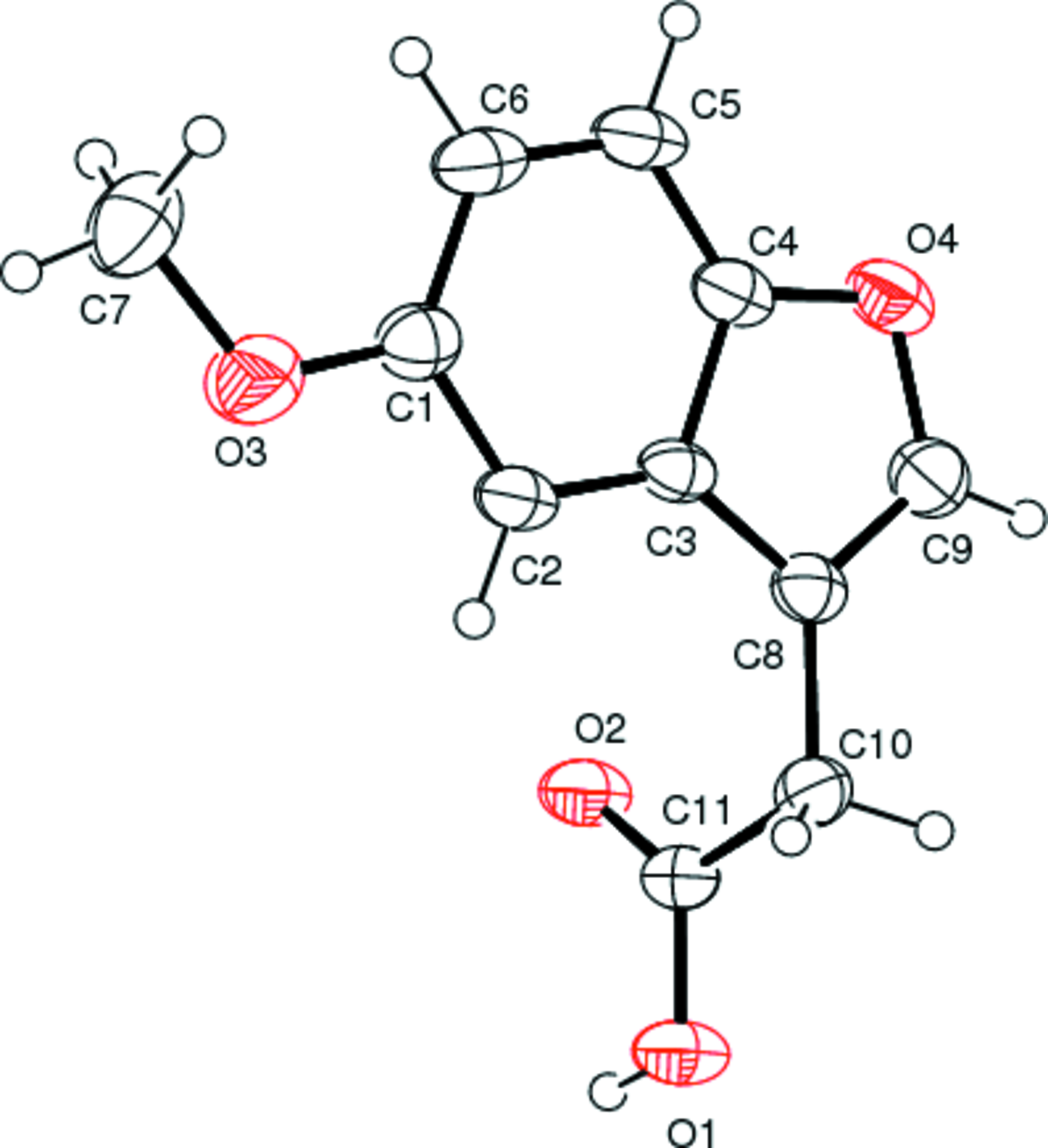

Supplement: Supplementary file 4 [file e-71-o1053-fig1.tif]
